# 1376. Cryptococcal Infection Presenting Solely as a Pleural Effusion

**DOI:** 10.1093/ofid/ofab466.1568

**Published:** 2021-12-04

**Authors:** Rodolfo M Alpizar-Rivas, Sally Chuang, Purba Gupta

**Affiliations:** University of Rochester, Boston, Massachusetts

## Abstract

**Background:**

Cryptococcal infections are frequently seen in immunosuppressed hosts. To date, few cases of cryptococcal infections presenting solely as pleural effusion have been described in liver transplant recipients. To our knowledge, this is the first case of cryptococcal pleuritis presenting with acute respiratory failure early post liver transplant.

**Methods:**

51- year old male with non- alcoholic cirrhosis complicated by chronic right hydrothorax underwent deceased donor liver transplantation with methylprednisolone induction. A week later, he developed acute respiratory failure requiring intubation. Pleural fluid was exudative with lymphocyte predominance; aerobic culture grew *C. neoformans*. Serum cryptococcal antigen was initially negative (prozone phenomenon was excluded) and subsequently turned positive titer 1:16. He was started on liposomal amphotericin and flucytosine, but developed acute kidney injury; induction therapy was changed to fluconazole with flucytosine for 2 weeks followed by fluconazole consolidation for 8 weeks. He remains on maintenance therapy. Donor serum cryptococcal antigen was negative, and recipients of other organs from the donor were clinically well.

**Results:**

Pleural effusions are common in cirrhotic patients with ascites from hepatic hydrothorax. Although rare, Cryptococcal infection can manifest as isolated pleural effusion. Our patient was diagnosed with Cryptococcal empyema early post-transplant, though likely had subclinical or latent infection pre-transplant; evaluation for donor-derived infection was negative. Diagnosis of isolated pleural disease may be missed if only serum Cryptococcal antigen is tested, as antigen may not be detectable. Diagnosis is mainly established by pleural fluid culture and may be delayed, as pleural fluid is not routinely cultured when effusions are attributed to hepatic hydrothorax. Cryptococcal antigen in the pleural fluid may have a better diagnostic yield.

**Conclusion:**

Cryptococcal infection should be considered in patients with cirrhosis and liver transplant recipients presenting with pleural effusion without any other abnormalities on chest imaging. Diagnosis may be missed if only serum cryptococcal antigen is used.

**Disclosures:**

**All Authors**: No reported disclosures

